# Inside the ‘Black Box’ of Pharmaceuticalisation: NICE, the Permissive Principle and the Case of Anticoagulants

**DOI:** 10.1111/1467-9566.70279

**Published:** 2026-10-16

**Authors:** John Abraham, Gowree Balendran

**Affiliations:** ^1^ Department of Medical Education Brighton and Sussex Universities Medical School (BSMS) Brighton UK; ^2^ Faculty of Medicine & Health Sciences Keele University Newcastle UK

## Abstract

Sociological interest in pharmaceuticalisation continues to grow, especially regarding the expansion of pharmaceutical use in society. This article presents the first major empirical study focusing on the directionality of pharmaceuticalisation by taking cost‐effectiveness assessment and regulation of anticoagulants in England by the National Institute of Health and Care Excellence (NICE) as a case study. During this century, anticoagulant therapy for the prevention of stroke and systemic embolism has changed direction profoundly away from warfarin towards newer and markedly more expensive direct oral anticoagulants. Drawing on established theoretical frameworks of pharmaceuticalisation, together with years of documentary research, interviews and observations of stakeholder meetings, it is demonstrated that this directional change cannot be adequately accounted for solely by reference to biomedicalism. Rather, such change must also be explained by key sociological factors, including the adoption of the permissive principle towards the newer drugs by the NICE regulatory system, pharmaceutical industry influence on governance processes, the ‘coincidence’ of expansion in diagnostic criteria for stroke risk with the introduction of the new direct oral anticoagulants, and the promotion of neoliberal patient choice and innovation by the regulatory state. Finally, the generalisability and policy implications of our findings for other therapeutic and institutional contexts are considered.

## 1 Introduction and Conceptual Framework

This article is about the pharmaceuticalisation of the prevention of stroke and systemic embolism (mobile blood clots) in patients with non‐valvular atrial fibrillation—a heart condition with elevated risk of blood clots. Pharmaceuticalisation was first substantively defined in sociology as: ‘the process by which social, behavioural or bodily conditions are treated, or deemed to be in need of treatment, with medical drugs by doctors or patients’ (Abraham 2009, 100). Subsequent theoretical discussions have focused on the scope of the concept, its relationship with medicalisation and how it broadly expresses expansion of pharmaceutical use in society (Abraham 2010; Bell and Figert 2012; Busfield 2010; Williams et al. 2011). Empirical investigations have included studies of anxiety/depression, cancer care, coronary heart disease, obesity, osteoporosis and sleeplessness, among others (Abraham and Sheppard 1999; Benavides 2025; Coveney et al. 2019; Davis 2015; Guillemot et al. 2025; Pollock and Jones 2015). Emphasis on the *growth* and/or *drivers of growth* in pharmaceuticals' diffuseness is striking, especially regarding pharmaceuticalisation as an alternative to lifestyle change, psychological intervention or surgery, though Abraham (2010, 611) notes the importance of ‘de‐pharmaceuticalisation’—the contraction of pharmaceutical prescription/use in favour of nondrug therapies. Yet, there has been little or no sociological research on the *directionality* of pharmaceuticalisation, such as how one drug therapy gives way to another pharmaceutical intervention. Although innovation studies typically describe the process by which industry develops new drugs and seeks to match them to markets ostensibly based on technical claims that they are superior to existing drugs, they do not examine how regulatory evaluations influence such pharmaceuticalisation.

Here, we present a microsociological analysis of how cost‐effectiveness regulatory decision‐making has dramatically altered the *direction* of pharmaceuticalisation of stroke prevention from treatment with warfarin to newer direct oral anticoagulants (DOACs) instead for patients in England with non‐valvular atrial fibrillation (NVAF) this century.^1^ The fundamental research question is: How did DOACs come to be deemed as recommended/preferable treatments to warfarin for patients with NVAF? In answering this, we provide the first major empirical study of how decision‐making within one of the world's most influential pharmaceutical cost‐effectiveness institutions relates to pharmaceuticalisation.

In line with Douglass (2023), we employ a combination of Abraham's (2002, 2009) concept of the ‘permissive principle’ and pharmaceuticalisation. Abraham and Davis (2009) defined the permissive principle as the tendency of regulatory agencies, under conditions of scientific uncertainty, to allow new drug products onto the market despite not meeting those agencies' own established standards of safety and/or efficacy. We extend its application to investigate if, under conditions of uncertainty about cost‐effectiveness, there is any tendency for regulatory institutions to recommend new drugs as cost‐effective for the healthcare system even when those recommendations do not meet the regulatory institutions' own established standards/findings about cost‐effectiveness. In comparison to a precautionary principle of technology development, utilising the permissive principle guides us to regulatory institutions' own standards/findings as a basis from which to determine whether the aforementioned change in direction of pharmaceuticalisation can be explained by biomedicalism or permissive regulatory decision‐making related to empirically identifiable sociological factors, perhaps involving industry influence, conflicts of interest, neoliberalism or regulatory‐state ideology of innovation. As Hogarth and Loblova (2022) note, health technology assessment is interconnected with market approval/licensing, clinical guidelines defining use and funding recommendations—all of which we consider in relation to key social forces involved, such as drug safety/efficacy regulators, the pharmaceutical industry, medical experts and perceived patient demand.

## 2 Background and Research Questions

Atrial fibrillation (AF) is the commonest cardiac arrhythmia (irregular heartbeat), affecting 60 million people worldwide and about 1.5 million in England, most of whom (over 70%) have non‐valvular atrial fibrillation (NVAF)—the condition in the absence of heart‐valve disease (Mahase 2023; Mbroh et al. 2025; Molteni et al. 2014). AF is associated with a fivefold increased risk of blood clots within the heart and consequent *ischaemic* stroke if those clots become detached and travel in the bloodstream to obstruct blood flow to the brain—thromboembolism (Stroke Association 2024). Anticoagulants reduce ischaemic stroke risk in patients with AF by inhibiting blood clotting. This is a delicate balance because excessive anticoagulation could cause dangerous/fatal bleeding, including haemorrhagic stroke and/or major gastrointestinal bleeding.

Warfarin, the first oral anticoagulant drug in clinical use since 1954, has a narrow therapeutic window (safest and most‐effective dose range), so doctors need to establish/adjust the correct dosage by monitoring patients' clotting characteristics (FDA Product Label 1954; Hogarth and Martin 2021). Crucially, successful treatment for patients, therefore, depends on whether warfarin therapy is well controlled or poorly controlled (determined by a blood test measurement, known as international normalised ratio or INR). It is estimated that, among patients with AF in the United Kingdom, about 44% are well controlled on warfarin (Macedo et al. 2015).

After 2010, new alternative oral anticoagulants entered the market, whose manufacturers claimed to be more convenient, cost‐saving innovations that could be administered as fixed oral doses without monitoring or dose adjustments (AstraZeneca 2003; Boehringer Ingelheim 2009). Commercial interests for pharmaceutical companies in these innovations are huge, with global market value over US$12 billion (Boehringer Ingelheim 2024; Daiichi‐Sankyo 2024; Johnson & Johnson 2025; Pfizer 2024). These became known as *direct* oral anticoagulants (DOACs), four of which were approved onto the UK market by the European Medicines Agency (EMA) and recommended as cost‐effective for use in the NHS during the 2010s by technology assessment (TA) committees of the National Institute for Health and Care Excellence—NICE^2^ (Table 1).

**TABLE 1:** Oral anticoagulants recommended by NICE for prevention of stroke and systemic embolism in NVAF.

Warfarin Used as an anticoagulant since 1954
Dabigatran Market‐approved by EMA in June 2011 Recommended by NICE (TA249) in March 2012
Rivaroxaban Market‐approved by EMA in September 2011 Recommended by NICE (TA256) in May 2012
Apixaban Market‐approved by EMA in September 2012 Recommended by NICE (TA275) in February 2013
Edoxaban Market‐approved by EMA in April 2015 Recommended by NICE (TA355) in September 2015

*Source:* NICE Technology Appraisal Guidance TA249, TA256, TA275 and TA355.

In just 3 years, 2015–2018, prescriptions for DOACs in England grew from 2.571 million to 9.317 million, whereas prescriptions for warfarin fell from 11.551 million to 8.243 million (Table 2). Prescriptions for DOACs had increased from less than a fifth of the ‘market’ to just over half, effectively displacing warfarin as the medication prescribed by doctors.

**TABLE 2:** Increasing use and cost of DOACs compared with warfarin.

Medicine	Number of prescriptions					Prescription costs				
	2015	2016	2017	2018	2023	2015	2016	2017	2018	2023
Dabigatran	366,000	415,137	458,103	478,179	301,322	£22,971,100	£20,893,245	£22,873,362	£24,152,154	£16,217,463
Rivaroxaban	1,537,700	2,528,799	3,306,572	3,921,741	4,317,002	£83,987,300	£121,130,897	£161,307,500	£194,482,896	£227,725,890
Apixaban	667,200	1,694,290	3,093,056	4,573,601	8,406,295	£37,268,200	£84,418,170	£150,397,309	£223,354,977	£335,372,320
Edoxaban	200	12,518	95,093	343,968	4,243,272	£14,500	£670,298	£4,637,669	£16,594,168	£213,733,695
DOACs % of total prescriptions	2,571,100 18%	4,650,744 30%	6,952,824 42%	9,317,489 53%	17,267,891 83%	£144,241,100	£227,112,610	£339,215,840	£458,584,195	£793,049,368
Warfarin	11,550,900 82%	10,789,270 70%	9,579,909 58%	8,243,486 47%	3,618,951 17%	£23,091,800	£17,554,051	£13,539,938	£8,854,504	£6,883,274

*Source:* Prescription Cost Analyses 2016–2019 and Prescription Cost Analysis 2024.

This came at considerable cost to the NHS—by 2018, the cost of DOACs combined reached £458M, whereas in the same year, warfarin's cost was under £9M. By 2023, DOACs comprised 83% of prescriptions, costing the NHS £793M, whereas warfarin constituted 17%, costing just £6.88 M (Table 2).

Even taking account of the additional blood tests/monitoring needed with warfarin compared with DOACs, warfarin remained cheaper; DOACs were between 25 and 80 times more expensive to prescribe than warfarin (Table 3). Based on assumptions that accept DOAC manufacturers' claims about monitoring requirements, warfarin users needed 6–12 blood tests per year, whereas DOAC users needed only one annually, effectively costing £17.45–£19.20 and £1.45–£1.60, respectively, in 2017 for blood tests/monitoring in a 28‐day treatment period (Connock et al. 2007, 33; NICE 2014a, 2014b). Hence, the overall 28‐day treatment costs for DOACs in 2017 were £50.45–£54.80, but only £18.11–£21.19 for warfarin, making DOACs at least 2.4–3.0 times more expensive (Table 3). Moreover, such quantifications leave aside arguments that more frequent monitoring can deliver health benefits for some patients so should not necessarily be considered solely as a cost.

**TABLE 3:** Comparative costs of DOACs and warfarin.

	Warfarin	Edoxaban (Lixiana)	Dabigatran (Pradaxa)	Rivaroxaban (Xarelto)	Apixaban (Eliquis)
Usual dose	3–9 mg ONCE daily (maintenance dose)	60 mg ONCE daily	150 mg TWICE daily	20 mg ONCE daily	5 mg TWICE daily
Costs (28 days)	£0.66–£1.99^a^	£49.00 (Not including Rebate)	£51.00	£50.40	£53.20

*Note:* Costs of oral anticoagulants for non‐valvular atrial fibrillation (adult > 18 years): Comparative costs: Each of the newer oral anticoagulants has a considerably higher acquisition cost than warfarin. When the cost of INR monitoring is taken into account, warfarin is likely to remain the least expensive option up‐front. Comparative cost‐effectiveness is not clear. If a DOAC is preferred and all other factors are equal, the DOAC with the lowest acquisition cost should be chosen.

^a^ 9 mg dose cost based on 1 mg + 3 mg + 5 mg tablets.

*Source:* Cambridgeshire and Peterborough Clinical Commissioning Group (2017). Costs of oral anticoagulants for non‐valvular atrial fibrillation. Available at https://www.cambridgeshireandpeterboroughccg.nhs.uk/easysiteweb/getresource.axd?assetid=11102&type=0&servicetype=1 (accessed 17th June 2019).

Evidently, the direction of pharmaceuticalisation of stroke prevention for patients with NVAF changed from being dominated by warfarin treatment to widespread prescription of DOACs, raising a number of subsidiary research questions. Can this be explained by what Abraham (2010) calls the ‘biomedicalism thesis’, which, as applied to this case study, is that the DOAC innovations offer real therapeutic improvements over warfarin, which justify their more expensive use on the NHS? Alternatively, can it be explained by intense profit‐driven industry promotion/marketing of new drugs directly to prescribing doctors, irrespective of whether the drugs are needed? Or by industry‐funded ‘disease‐awareness campaigns’ aimed at persuading patients to seek treatments from their doctors? Or by how general practitioners, local primary‐care trusts or hospitals respond to NICE guidelines? These are explanations which many scholars have identified in accounting for pharmaceuticalisation more generally (Acharya and Hamis 2024; Busfield 2010; Douglass and Calnan 2021; Spyridonidis and Calnan 2011; Saghy et al. 2021).

Although these alternative explanations may be very important *after* a pharmaceutical is recommended by NICE, new medicines cannot enter the market unless/until they are approved as safe and effective by government drug product regulatory agencies—the UK's Medicines and Healthcare products Regulatory Agency (MHRA) and, before Brexit, the EU's EMA (Abraham and Lewis 2000). Furthermore, if market‐approved, they are not recommended for prescription by doctors in England's NHS unless approved by NICE as cost‐effective. As the NHS represents the vast majority of England's prescription medicine market, the role of government, and of NICE in particular, is crucial. It is very difficult, if not impossible, for pharmaceutical companies to promote drugs to NHS doctors if NICE has recommended that they should not be purchased by the NHS.

Notwithstanding pharmaceutical companies' enormous *potential* power in promoting their products directly to NHS doctors and indirectly to patients, and the agency of local hospitals and clinical practices in implementing NICE guidelines, evidently a methodologically pre‐eminent research question must be about NICE's regulation of DOACs. Instead, or as well as biomedicalism, industry promotion, doctors' prescribing and/or patients' expectations, might the aforementioned change in direction of pharmaceuticalisation be significantly explained by sociological factors inside the black box of NICE governance, such as neoliberalism, industry influence, conflicts of interest and/or ‘regulatory‐state ideology’ of permissiveness towards the interests of industrial innovation, irrespective of real cost‐effectiveness for the NHS and public health?

## 3 Research Design and Methods

Sociological studies of NICE and other national drug cost‐effectiveness regulatory agencies have examined how such institutions manage uncertainty, transparency and stakeholders during public controversies over availability of drugs for multiple sclerosis and Alzheimer's disease, but those studies do not engage with pharmaceuticalisation (Calnan et al. 2017; Crinson 2004; Csanadi et al. 2019; Ozieranski and King 2016). As this is the first empirical investigation of substantive conceptual frameworks of pharmaceuticalisation in relation to cost‐effectiveness assessment and regulation, a case‐study design was adopted to enable in‐depth exploration of the explanatory factors of pharmaceuticalisation involved. Our research sought answers via literature review, freedom‐of‐information requests to access unpublished data, documentary analysis, observation and ‘expert’ interviews (Bogner et al. 2009). NICE was the main ‘spatial’ field domain, whereas warfarin and DOACs were the main ‘technical’ domains. The intersection of these domains formed the primary microsociological research focus. Particular attention was given to NICE's Clinical Guideline CG180, published in 2014, which provides guidance for the NHS on management of atrial fibrillation, and to NICE's Technology Appraisal Committees (TACs) and Evidence Review Groups (ERGs) for the four DOACs involved, namely, apixaban, dabigatran, edoxaban and rivaroxaban (NICE 2014b).

A systematic review of relevant social science, medical, pharmaceutical and regulatory literature pertaining to NICE, warfarin and DOACs from the beginning of this century was conducted together with comprehensive analysis of NICE websites, annual reports and minutes/reports of NICE TACs and ERGs. Key search terms included ‘apixaban’, ‘cost‐effectiveness’, ‘dabigatran’, ‘edoxaban’, ‘efficacy’, ‘DOACs’, ‘EMA’, ‘FDA’,^3^ ‘MHRA’, ‘NICE’, ‘rivaroxaban’, ‘safety’ and ‘warfarin’.

Documentary information about DOAC clinical trials was obtained from the medical/technoscientific literature, manufacturers' submissions to NICE, industry publications (e.g., *Association of the British Pharmaceutical Industry News*), web‐based sources (e.g., clinicaltrials.gov) and periodicals on the pharmaceutical sector, such as *Scrip: World Pharmaceutical News*. We obtained regulatory‐review documents on DOAC products' safety and efficacy generated by the EMA, MHRA and FDA. The MHRA and EMA approved and/or maintained DOACs as safe and efficacious on the UK market, which informed NICE's cost‐effectiveness assessment for the NHS. Data regarding the FDA's regulation of the same DOACs were also obtained because the FDA allows extensive public access to information submitted to it by pharmaceutical manufacturers, providing helpful contextual insights.

Following purposive‐sampling interview methodology, interviewees were nonrandom ‘informants’ selected for expert knowledge about DOACs and/or involvement in pharmaceutical governance, rather than as demographic or institutional ‘representatives’ per se. Suitability for selection was determined from documentary analysis and observation at relevant multistakeholder events. Eighteen semi‐structured interviews were conducted—seven with key NICE medical/scientific personnel, including members and former expert advisers of ERGs and TACs for DOACs, as well as members of the Guideline Development Group (GDG) for CG180. Representatives from the pharmaceutical industry and the UK Department of Health were interviewed, as were physicians with specialist experience of prescribing DOACs, academic scientists who had published research on DOACs and two former FDA scientists who had reviewed the product safety and efficacy of DOACs in the United States.

Interviews took place during 2015–2019 and were completed with ethical approval and informed consent, audiotaped and transcribed. Meanwhile, 13 multistakeholder meetings were observed, including NICE Fora, annual conferences and workshops relating to NICE's pharmaceutical assessments. After observation, detailed field notes were immediately prepared. Interview and field note data were subsequently thematically coded for analysis.

All data were analysed according to the research objectives. Interviews were analysed using manual qualitative content analysis based on inductive and deductive themes (Schreier 2014). Triangulation between data sources was employed to strengthen validity of our empirical findings (Guion 2002). For example, to investigate the significance of the biomedicalism thesis in explaining directional change in pharmaceuticalisation of stroke prevention, we analysed documentary data (clinical trial and regulatory evidence) about DOACs' safety and efficacy (compared with warfarin) in conjunction with regulators' interview responses about such matters. Conversely, the exploratory nature of interviews/observations sometimes evoked explanatory themes within the pharmaceuticalisation process, such as ‘patient choice’ or ‘industry influence’, which could be confirmed by documentary data analysis. Given our primary interest in sociological institutions/processes/explanations, we have anonymised individual actors.

## 4 Biomedicalism Considered: Comparative Efficacy, Safety and Benefit‐Risk Profile

If DOACs were clearly shown to have superior efficacy and/or safety to warfarin, then NICE's recommendation to the NHS that they were/are cost‐effective (and consequent re‐direction of pharmaceuticalisation), despite being more expensive than warfarin, could be explained almost entirely by biomedical progress. Manufacturers chose to compare the efficacy of each of the DOACs with warfarin *not in superiority trials* but rather in large international multicentre ‘non‐inferiority’ trials defined by the International Conference on Harmonisation of Technical Requirements for Registration of Pharmaceuticals for Human Use (ICH)^4^ as follows:

Clinical trials designed to demonstrate efficacy of a new drug by showing that it is similar in efficacy to a standard agent [warfarin] have been called ‘equivalence’ trials. Most of these are actually non‐inferiority trials attempting to show that the new drug [DOAC] is not less effective than the control [warfarin] by more than a defined amount.

(ICH 2001, 12)

Thus, the key comparative efficacy trials submitted to the EMA and FDA between 2005 and 2015 to gain product approval onto the EU (then including the United Kingdom) and US markets were designed to demonstrate *at best* that DOACs were equivalent to warfarin in effectiveness. Details of the key trials are provided in Table 4.

**TABLE 4:** Summary of clinical trials and regulation for quality, safety, efficacy and cost‐effectiveness for the DOACs.

Direct oral anticoagulant (DOAC)	Clinical trial	EMA approval (marketing authorisation)	NICE guidance
Dabigatran			
Dabigatran etexilate for prevention of stroke and systemic embolism in people with atrial fibrillation	RE‐LY Unblinded, noninferiority, randomised controlled trial (18,113 patients enrolled in 951 centres in 44 countries)	June 2011 Prevention of stroke and systemic embolism in adult patients with atrial fibrillation. Sequential dosing regimen 110 mg twice a day (≥ 80 years) 150 mg twice a day (< 80 years)	March 2012 (TA249)
Rivaroxaban			
Rivaroxaban for prevention of stroke and systemic embolism in people with atrial fibrillation	ROCKET–AF Multicentre, double‐blind, noninferiority, randomised controlled trial (14,264 patients enrolled in 1178 sites in 45 countries) Defective warfarin monitoring device used, but claimed by manufacturers and accepted by regulators not to have invalidated trial	September 2011 Prevention of stroke and systemic embolism in adult patients with NVAF with one or more risk factors, such as congestive heart failure, hypertension, age ≥ 75 years, diabetes mellitus, prior stroke or transient ischaemic attack 15 mg or 20 mg once a day	May 2012 (TA256)
Apixaban			
Apixaban for preventing stroke and systemic embolism in people with NVAF	ARISTOTLE International, multicentre, double‐blind, noninferiority, randomised controlled trial (18,201 patients enrolled at 1034 clinical sites in 39 countries).	September 2012 Prevention of stroke and systemic embolism in adult patients with NVAF with one or more risk factors 5 mg twice a day 10 mg twice a day	February 2013 (TA275)
Edoxaban			
Edoxaban for preventing stroke and systemic embolism in people with NVAF	ENGAGE AF‐TIMI 48 International, multicentre, double‐blind, noninferiority, randomised controlled trial (21,105 patients enrolled at 1393 sites in 46 countries). Questionable efficacy compared with warfarin in patients with normal renal function	April 2015 Prevention of stroke and systemic embolism in adult patients with NVAF with one or more risk factors 60 or 30 mg 30 or 15 mg	September 2015 (TA355)

The primary efficacy goal (endpoint) of anticoagulant treatment is to prevent strokes and systemic embolism due to excessive blood clotting—*ischaemic* strokes. The clinical trials also collected (safety) endpoint data on potential adverse effects of the anticoagulants, such as haemorrhagic strokes (caused by excessive bleeding), gastrointestinal bleeding and heart attacks, among others. Based on clinical trial data, all four DOACs were approved by the EMA as noninferior to warfarin in preventing strokes^5^ and systemic embolism (Connolly et al. 2009; Patel et al. 2011; Granger et al. 2011; Giugliano et al. 2013). Only the twice‐daily 150 mg, but not the 110 mg, dose of dabigatran was found to be superior to warfarin in this regard.^6^ However, FDA scientists concluded that dabigatran‐150 mg showed ‘efficacy, but not superiority over warfarin’ *when patients were well controlled on warfarin* (Beasley and Thompson 2010, 14–15, 165; Spackman et al. 2011, 9).

Moreover, compared with warfarin, there were 63 more cases of major gastrointestinal bleeds with dabigatran‐150 mg, of which 38 were life‐threatening, but only 30 fewer ischaemic strokes and 33 fewer haemorrhagic strokes (Boehringer Ingelheim 2011a, 128, 222). Thus, for every additional stroke prevented by dabigatran‐150 mg, there was an additional major gastrointestinal bleed induced, often life‐threatening. Similarly, clinical trials with apixaban showed that it was associated with fewer haemorrhagic strokes than warfarin but more serious gastrointestinal bleeds (NICE 2013, 8–13).

The key dabigatran trial submitted to regulators (EMA, FDA and NICE), code‐named RELY and sponsored by Boehringer Ingelheim, was a large unblinded trial (Table 4; Connolly et al. 2009; Spackman et al. 2011). FDA scientists warned against granting a superiority claim to dabigatran‐150 mg over warfarin based on results from a single unblinded trial^7^ because only 33 adverse events would be required to reverse the supposed superiority, whereas 235 patients on dabigatran‐150 mg had prematurely discontinued treatment in the trial (Beasley and Thompson 2010, 48, 62). Furthermore, absolute differences between dabigatran and warfarin for most efficacy and safety endpoints were *less than 1%*, which Moore et al. (2014, 1) argued were so small, from a huge multicentre unblinded trial, that their clinical relevance was/is unclear, even though some of the small differences were statistically significant because of the large sample size.

Thus, apart from the manufacturers and their paid consultants, there was a consensus among product regulators (EMA and FDA) and independent medical scientists that DOAC clinical trials implied equivalence or noninferiority to warfarin, but *not* superiority. Indeed, in the manufacturer's own key edoxaban clinical trial (code‐named ENGAGE), warfarin was associated with fewer ischaemic strokes than edoxaban in patients with normal kidney function to such an extent that the FDA warned against using edoxaban in such patients in the United States (Blank et al. 2015, 2–5; Greenhalgh et al. 2015, 32), whereas the key rivaroxaban trial was found to have been conducted using a defective INR monitoring device throughout the trial—believed by several independent medical experts to have invalidated the trial results (Balendran and Abraham 2026).

Beyond efficacy/safety data, DOAC manufacturers claimed that a significant convenience and cost advantage derived from the biomedical nature of their drugs was that they did not require dose monitoring/adjustment (AstraZeneca 2003; Boehringer Ingelheim 2009). As previously discussed, there is truth in this argument, but it should not be exaggerated. There is evidence, even before approval, that monitoring exposure to DOACs might be needed for patients with an increased risk of bleeding (Cohen 2014; Douxfils et al. 2015; Reilly et al. 2014, 328). When the EMA and FDA approved dabigatran, those regulatory agencies and medical scientists working for Boehringer Ingelheim were aware of potential safety problems with the drug if blood levels were not measured/monitored (Moore et al. 2014; Reilly et al. 2014).

Even if one accepts that DOACs confer a beneficial advantage over warfarin in not needing dose monitoring and adjustment, there are disadvantages to set against this. For decades, there has been an antidote (vitamin K), which can reverse the effects of warfarin if necessary (e.g., uncontrollable bleeding), but all the DOACs were approved onto the market *years* before any antidotes (idarucizumab and andexanet alfa) were available for them: apixaban, 6 years; dabigatran, 3 years; edoxaban, 4 years; and rivaroxaban, 7 years (EMA 2015; Link 1959; NHS Specialist Pharmacy Service 2019; NICE 2016). This placed patients at additional risk of bleeding to death compared with warfarin until 2019, when antidotes became available. Additionally, official estimates of relative costs per patient to the NHS of DOAC antidotes are exorbitantly high: £2400–£4800 for idarucizumab and £15,000 for andexanet alfa, but only £3.78–£15.12 for vitamin K injections to reverse the effects of warfarin (MIMS 2016; NICE 2016, 12–13; NICE 2020).

Relatedly, it is not obvious that DOACs are safer than warfarin. By 2016, DOACs only accounted for 30% of prescriptions (Table 2), but over 70% of reported deaths (Table 5). Our argument is not that DOACs were/are less efficacious or safe than warfarin, but merely to point out that, during the 2010s, when NICE came to assess their *cost‐effectiveness*, there was *not* convincing evidence overall that they were *biomedically superior* to warfarin.

**TABLE 5:** Safety profile for warfarin and DOACs in the United Kingdom.

Medicine	Number of deaths				
	November 2008–March 2011	November 2008–August 2016	November 2008–May 2017	November 2008–March 2018	November 2008–May 2019
Dabigatran	4	141	147	158	165
Rivaroxaban	9	246	292	346	400
Apixaban	—	80	116	170	240
Edoxaban	—	2	3	9	18
All DOACs	13	469	558	683	823
Warfarin	37	200	235	263	299

*Source:* MHRA Interactive Drug Analysis Profiles (warfarin 2008, 2011, 2018 and 2019; dabigatran 2011, 2018 and 2019; rivaroxaban 2011, 2018 and 2019; apixaban 2018 and 2019; edoxaban 2018 and 2019) and MHRA Drug Analysis Prints 2016 and 2017 available at http://www.mhra.gov.uk/drug‐analysis‐prints/.

## 5 Permissive Cost‐Effectiveness Assessment at NICE

NICE's cost‐effectiveness assessment was conducted by one of its Technology Appraisal Committees (TACs) with co‐opted expert advisers known as clinical specialists. Commissioned academic groups, known as Evidence Review Groups (one for each DOAC), reviewed the evidence submitted to NICE by the manufacturers to inform the TAC. After 2008, after criticisms from some pharmaceutical companies, patient organisations and politicians, NICE introduced single technology assessments (STAs) instead of the previous system of multiple technology assessments (MTAs) to accelerate/streamline the review process in keeping with other regulatory trends of the ‘neo‐liberal era’ (Abraham and Balendran 2025; Davis and Abraham 2013, 2). The MTA process had given Evidence Review Groups (ERGs) time to develop their own independent analyses of drugs' cost‐effectiveness data, whereas under STA, the ERG's role was diminished to a review of manufacturers' data. That change gave greater prominence to industry data submissions within NICE, despite an understanding that manufacturers' results were often more optimistic about the value of new drugs than those of the independent ERGs under MTA (Claxton et al. 2025).

In this structural institutional context, DOACs underwent STA assessment at NICE. The ERG comparing warfarin with dabigatran concluded:

Considering the patients able to maintain INR within the target range [i.e. *well‐controlled*], …warfarin was the most cost‐effective intervention for patients. …the cost‐effectiveness of dabigatran differs by severity and …it is not cost‐effective for patients who can maintain adequate INR levels [i.e. *well‐controlled*].

(Spackman et al. 2011, 16, 122–123)

The implication of this might be that dabigatran should be recommended for second‐line prescription on the NHS for only patients who were not well controlled on warfarin. That would have been a smaller market for the DOAC, contrary to the commercial interests of the manufacturer, who disputed the ERG's conclusions (Boehringer Ingelheim 2011b, 16). One of NICE's clinical specialists for the TAC, who had been a clinical investigator on the manufacturer's RELY trial, a consultant for the manufacturer and had declared conflicts of interest, also argued that dabigatran should not be recommended for second‐line use because it was associated with a lower risk of haemorrhagic strokes than warfarin, but without acknowledging (as we noted above) that the risk was offset by an equally higher risk of life‐threatening gastrointestinal bleeding (NICE 2011b, 2012b, 23). Ultimately, NICE (2012b, 2014b) sided with the manufacturer and this specialist, rather than its own ERG, and recommended dabigatran for *all* NHS patients with NVAF, *including* those who were well controlled on warfarin.

Similarly, NICE (2012a, 2014b, 2015) recommended rivaroxaban and edoxaban to the NHS as cost‐effective for stroke prevention in *all* NVAF patients with one or more of the following risk factors: age 75 or older; prior stroke/transient ischaemic attack; congestive heart failure; hypertension; or diabetes. Yet rivaroxaban was much more expensive than warfarin, and the FDA had already found from the manufacturer's clinical trials that the drug was less effective than *well‐controlled* warfarin:

….the data do not convincingly demonstrate that rivaroxaban is as effective in preventing strokes and systemic emboli as warfarin when warfarin is used skilfully.

(Beasley et al. 2011, 14)

In addition, NICE's ERG on rivaroxaban, together with other stakeholders, was concerned that the manufacturer's large key multinational trial comparing the drug with warfarin might not be generalisable to UK clinical practice because only 0.2% of patients on the trial had a low level of risk factors compared with 20%–75% of the UK population with atrial fibrillation at similar risk for whom warfarin was prescribable (NICE 2012c, 21). Nonetheless, ultimately, NICE was willing to be persuaded by the manufacturer that the trial findings were generalisable to the United Kingdom (NICE 2012c, 21). NICE's TAC for rivaroxaban was advised by patient groups and two clinical specialists, one of whom was nominated by the manufacturer. Both specialists declared considerable personal financial interests in the manufacturer (NICE 2011a, 4–5).

Edoxaban, too, was significantly more expensive than warfarin, whereas NICE (2015, 20–21) itself acknowledged that this DOAC was merely ‘as clinically effective’ as well‐controlled warfarin.

Hence, by NICE's own conclusions, edoxaban was less cost‐effective than well‐controlled warfarin. Regarding edoxaban's higher rate of ischaemic strokes than warfarin in patients with normal kidney function, edoxaban's manufacturer told NICE that this might be explained by ‘random variability’ due to ‘some unidentified sources of bias’ in ENGAGE (Daiichi‐Sankyo 2015, 80). While noting the FDA's warning against approving edoxaban for patients with normal kidney function, NICE's TAC, advised by its clinical specialist, accepted the company's hypothetical explanation (Greenhalgh et al. 2015, 81–82; NICE 2015, 21). That Committee was advised by only one clinical specialist who was nominated by Daiichi‐Sankyo, edoxaban's manufacturer, and had multiple financial links (consultancy fees and research funding) with the manufacturer for several years (Bakhai et al. 2013, 9; Bakhai 2015, 9).

Only NICE's (2013, 20–22) promotion of apixaban as cost‐effective for the NHS was consistent with its own view that the DOAC was ‘clinically more effective’ than warfarin due to fewer haemorrhagic strokes—a generous interpretation of the key clinical trial (code‐named ARISTOTLE), which did not find any statistically significant difference between the two drugs in preventing ischaemic stroke or systemic emboli. Even apixaban's advantage over warfarin regarding all strokes (ischaemic and haemorrhagic) amounted to just 0.6%, which some FDA scientists regarded as fragile because they estimated that as many as 15% of patients who had discontinued medication were not followed up (Marciniak et al. 2016, 258).^8^ Two out of the three clinical specialists advising NICE about apixaban declared conflicts of interest, one being manufacturer‐nominated (NICE 2012a).

These findings imply that there was considerable, application of the permissive principle within NICE's final decisions to recommend DOACs as cost‐effective for the NHS and that NICE's decision‐making was attended by frequent conflicts of interest which militated in favour of permissiveness, significantly enabling DOAC pharmaceuticalisation.

## 6 NICE Guidance: Pharmaceuticalising Stroke Risk and Prevention

The Guideline Development Group (GDG) for NICE's Clinical Guideline CG180 considered stroke prevention to be the single greatest priority in atrial fibrillation and sought to ‘improve anticoagulant uptake nationwide’ (Cowan et al. 2015, 1). CG180 achieved a ‘paradigm change’ because it advocated anticoagulation as the norm for all patients, except a minority with the lowest risk of stroke (Cowan et al. 2015, 2). As one GDG member put it:

….It became almost that the default for any patient with atrial fibrillation should be anticoagulation other than the very low risk patients …. there has been a change in thinking …to contribute in the improvement in anticoagulation uptake.^9^

Specifically, ‘based on the experience and opinion of the GDG’, CG180 advised that all patients with NVAF should be reviewed ‘at least annually’ for stroke and bleeding ‘risk factors’ that were used to develop ‘risk stratification tools’ to predict stroke risk in prophylactic treatment (Gage et al. 2001; Jones et al. 2014; Lip 2013; Lip et al. 2010; NICE 2014b). Moreover, CG180 recommended that NHS health professionals use new stroke risk‐stratification tools developed by a GDG member—a professor funded by pharmaceutical companies as a clinical investigator for all four DOACs, who withdrew from two GDG discussions about risk‐stratification tools due to conflicts of interest. Those risk‐stratification tools widened the definition of stroke risk by incorporating vascular disease and expanding the age range of patients from over‐75s to over‐65s (NICE 2014b, 113, 164). Previous stroke risk‐stratification tools had focused on treating only high‐risk patients. CG180 shifted towards the identification of patients at very low risk, who did not need anticoagulation, followed by prophylactic anticoagulant treatment for all other patients (NICE 2014b, 143). Relatedly, NICE's CG180 also recommended that elderly patients with AF should not be denied anticoagulants solely because of ‘excessive emphasis’ on falling as a risk factor for bleeding (NICE 2014b, 164–65).

In theory, these NICE recommendations facilitated expansion in the prescription of *all* anticoagulants for stroke prevention in NVAF, including DOACs *and* warfarin. However, prescription growth occurred disproportionately towards DOACs because NICE's recommendations came when DOACs were emerging as new drugs on the market with maximum promotional efforts to doctors by their manufacturers—the latter becoming possible after NICE's permissive recommendation to the NHS that DOACs were more cost‐effective than warfarin for all patients. Thus, application of the permissive principle and pharmaceuticalisation of stroke risk, attended by conflicts of interest, acted symbiotically to amplify directional change towards DOAC prescription.

## 7 Industry Influence, Neoliberal Patient Choice and the Ideology of Innovation

NICE’s Guideline CG180 recommends each DOAC as ‘an option’ for reducing the risk of stroke and systemic embolism in patients with NVAF (NICE 2014b, 50–51). This raises the question of which option to choose. According to some members of the Guideline Development Group (GDG) for CG180, there was ‘division’ within the GDG because one member felt that warfarin should be recommended in preference to DOACs due to insufficient evidence on DOACs' clinical effectiveness, though NICE's overall procedural policies meant that such matters were determined by NICE's Technology Appraisal Committee's decision and could not be considered within the guideline:

….what the guideline had to do was position warfarin and there was a lot of debate at the time. …even though one of the members of the Committee *didn’t* like the idea that the DOACs should be used so freely without more experience, saying: …‘I think you should really be putting predominant voice to warfarin—it’s a cheaper drug, we have experience with it’, the [project team] members from NICE were saying it didn’t matter because their position [on cost‐effectiveness] is defined by the technology appraisal[s] and we can’t go against the technology appraisal[s] [which] …had defined their position^10^ [emphasis in original].

….the scope [of CG180] did not include [the effectiveness of] DOACs. DOACs were a black box. They were going to be determined by these other technology appraisals …We did not address the issue of: ‘Are DOACs better than warfarin?’ So, it was not considered in the 2014 guideline. We were *absolutely* clear that patients should be given a choice^9^ [emphasis in original].

Evidently, the permissive cost‐effectiveness decision‐making about DOACs by the Technology Appraisal Committee procedurally proliferated to the GDG, which not only reproduced it but further expanded it via their neoliberal ideologies of patient choice and innovation.

Specifically, a key output from the GDG's work on CG180 was NICE's endorsement of a decision‐support tool to ‘inform patient choice’. CG180 recommends to NHS prescribers: ‘Discuss the options for anticoagulation with the person and base the choice on their clinical features and preferences’ (NICE 2014b, 143). Alongside this, NICE also simultaneously published a decision aid, now part of an online decision‐support tool, to facilitate decision‐making and inform patients' choice of anticoagulant (Haslam 2014). The contents of the decision aid were constructed by a collaboration between NICE, Keele University and the manufacturers of DOACs (Keele University 2016; NICE News 2015). Although NICE guidance promotes DOACs as options to warfarin, the emphasis on patient choice operates, via the decision‐support tool, to influence the prescription of DOACs *in preference* to warfarin, as a member of the GDG for CG180 acknowledged:

If you’ve seen the tool, it’s…going into the advantages or disadvantages of DOACs with respect to warfarin. So, the guidelines said you should be considering DOACs and you should be talking to your patients and the decision is theirs. The implementation tool sat alongside the guideline. I guess that…more *specifically* favoured DOACs…that *does* go some way to recommending DOACs [emphasis in original]…and I think that *any patient reading it would be more likely to consider a DOAC than warfarin* ^9^ [emphasis added].

For example, the decision aid emphasises the need for more frequent blood tests when taking warfarin compared with DOACs but makes no mention of uncertainties surrounding rivaroxaban due to defective monitoring devices during its trial or about edoxaban's higher rate of ischaemic strokes in patients with normal kidney function (NICE 2014a). Although patients were being persuaded to consider a DOAC rather than warfarin by the decision aid, NHS clinical commissioning groups were also urged in that direction by the NICE Implementation Collaborative, involving NICE, the life sciences/pharmaceutical industry, healthcare professional organisations and NHS senior management, to support a ‘culture shift’ within the NHS in favour of innovation (NICE Implementation Collaborative 2013, 2; ABPI [Association of the British Pharmaceutical Industry] 2013):

…simultaneously with the guideline, there was another initiative going on in NICE…to address…that the uptake of DOACs across the country was very patchy and some commissioning groups were discouraging the use of DOACs for reasons of costs.^9^

In particular, the NICE Implementation Collaborative (2014) recommended ways in which local GP practices could set up agreed protocols across primary and secondary care for initiating DOACs (Haslam 2014). As a GDG member explained, emphasis on patient choice in CG180 enabled DOACs to be prescribed if DOACs were preferred by patients, *despite* evidence that well‐controlled warfarin was more cost‐effective (Spackman et al. 2011, 122–123):

…they [NHS clinical‐commissioning groups] have got difficulty balancing their budgets and…there was reasonable evidence in the early DOAC trials that, if you had good anticoagulant control, that warfarin was as effective as DOACs in stroke prevention…I think that was contrary to the 2014 guideline, which was saying the *patient* should choose and they should have the option to go for DOACs or warfarin, rather than the commissioning group dictate that they [patients] must start warfarin first.^9^

Thus, the GDG hoped that, by promoting equality of access to DOACs, CG180 would end local commissioning pathways that required patients on warfarin to show poor control before a DOAC could be considered (Cowan et al. 2015). We refer to this as ‘neo‐liberal patient choice’ combined with an ‘ideology of innovation’ because it prioritises maximisation of individual choice between (anticoagulant) products in a pseudo‐marketplace over the collective interests of public health. The ideologies of neoliberal patient choice and innovation proliferated the permissive principle because NICE is supposed to prioritise the interests of public health, but they promoted DOAC pharmaceuticalisation in the commercial interests of manufacturers.

As well as involvement in shaping NICE's recommendations to NHS prescribers and patients about DOACs, the pharmaceutical industry also had financial links with many members of the GDG for CG180. The Group comprised four cardiologists, two GPs, one geriatrician, one A&E consultant, two cardiology specialist nurses, one pharmacist and two patient members, as well as two co‐opted expert advisers—a cardiothoracic surgeon and a haematologist (NICE Appendices 2014, 11–12). Only two (15%) of the 13 GDG members declared no conflicts of interest, whereas eleven (85%) declared interests, of which eight (61.5%) declared personal financial interests for payments received from the pharmaceutical/life sciences industry. Both expert advisers also declared personal pecuniary interests for consultancy work with pharmaceutical or medical device companies with an interest in CG180 (NICE 2008; NICE Appendices 2014, 15–31).

## 8 Discussion

The biomedicalism thesis cannot adequately explain the directionality of pharmaceuticalisation of stroke prevention in non‐valvular atrial fibrillation because there is little or no evidence that DOACs were superior to well‐controlled warfarin, merely noninferior. Hence, some other (sociological) explanations for such pharmaceuticalisation must be involved.

NICE's analyses found that three of the four DOACs were less cost‐effective than well‐controlled warfarin. Regarding the fourth, apixaban, any proclamation that it confers a significantly greater advantage than warfarin is ‘fragile’, according to FDA scientists who reviewed apixaban. Yet NICE recommended all four DOACs to the NHS in England as cost‐effective for *all* patients with NVAF, even though they were much more expensive than warfarin. In so doing, for at least three DOACs, NICE applied the permissive principle by recommending them for patients who were, or could be, well controlled on warfarin, despite having established by their own standards and analyses that the prescription of those DOACs was not as cost‐effective as well‐controlled warfarin.

Our findings here do not imply that DOACs should have been denied marketing approval or withheld from NHS patients but rather draw attention to *how* they were recommended to the NHS. An alternative pathway for NICE, based on its own analyses, could have been to recommend DOACs to the NHS as cost‐effective *when/if* patients could *not* be well controlled on warfarin. Such second‐line drug designation for DOACs would have been in the interests of the NHS and wider public health by preserving funds to be spent elsewhere on health but would have substantially shrunk the market for manufacturers, constrained total patient choice and curbed the ‘uptake’ of ‘innovation’. The pharmaceutical manufacturers argued against second‐line status and were supported by clinical specialists, often with financial links to the manufacturers involved. NICE's regulatory processes allowed such industry persuasion and patronage within its policies despite requiring experts/advisers/specialists to declare conflicts of interest with manufacturers transparently. Such policies imply institutional acknowledgement of a *risk* of permissive bias derived from conflict of interest (Abraham 1995; Lexchin and O'Donovan 2010). An individual's conflict of interest does not necessarily imply any impropriety, but our study suggests that risk can be high and insufficiently mitigated merely by requesting transparent declarations.

The outcome of such permissiveness was that it paved the way for manufacturers to vigorously promote DOACs to NHS doctors for all patients with NVAF, thus enabling the aforementioned re‐direction of pharmaceuticalisation. The significance of this for society and the NHS is underlined by the fact that, in the United Kingdom, the majority of DOAC prescriptions are for *lifelong* stroke‐risk prophylaxis in patients with NVAF. Such pharmaceuticalisation unnecessarily and chronically challenged healthcare systems' budgets at the expense of other more cost‐effective health interventions.

This was amplified by NICE's pharmaceuticalisation of stroke risk and prevention via widened diagnostic criteria for patients with AF to be prescribed anticoagulants, extended from high‐risk patients to all patients, except those at very low risk. Although this served to increase the uptake of anticoagulants (including warfarin) in stroke prevention and may be beneficial for some patients, the timing coincided with opportunities for maximum industry promotion of the new DOACs. Consequently, such pharmaceuticalisation expanded anticoagulant uptake mostly in the direction of DOACs.

Although much of NICE's adoption of the permissive principle towards DOACs was consistent with the commercial interests of the pharmaceutical industry, NICE was also committed to making DOACs available to increase *all* patients' choice of anticoagulants and to foster ‘innovation’. However, that ‘choice’ and ‘innovation’ was shaped by a framework involving the industry (including DOAC manufacturers) which tended to persuade patients and GP clinical practices to favour DOACs over warfarin. Whether such a neoliberal choice would be good for patients who were or could be well controlled on warfarin is debatable, but it was inconsistent with the collective interests of the NHS and public health because NICE had found that well‐controlled warfarin was more cost‐effective than DOACs. NICE's nurturing of such neoliberal patient choice and ideology of innovation further facilitated the pharmaceuticalisation of stroke prevention in the direction of DOACs and away from warfarin. There can be little doubt that such directionality was subsequently magnified by the manufacturers of DOACs who, empowered with NICE's seal of approval, vigorously promoted their own products to NHS prescribers.

## 9 Conclusion

Our findings show how cost‐effectiveness assessment and regulation—a hitherto neglected phenomenon in discussions of pharmaceuticalisation—can crucially influence the direction of pharmaceuticalisation well beyond a scientistic biomedical explanation. Our case study challenges the pervasive pharmaceutical policy narrative that NICE is a barrier to the adoption of expensive innovative pharmaceuticals. Rather, it supports the proposition (previously reserved for drug *product* regulatory agencies) that an important driver of pharmaceuticalisation is a ‘regulatory‐state ideology’ of permissiveness towards the interests of industrial innovation. We found that ideology to be attended by some direct industry influence, frequent expert conflicts of interest and a neoliberal conception of patient choice overriding cost‐effectiveness for the NHS and wider public health.

More broadly, our study shows how concepts and tools of medical sociology can be utilised to explain why pharmaceuticalisation takes particular form and direction down to the fine grain of distinctive therapeutic areas. Specifically, it shows how studies of pharmaceuticalisation can go beyond descriptions of broad ‘drivers’ to shed some evaluative light on how well the particular pharmaceuticalisation process in question serves the interests of public health.

Some evidence suggests that our case‐study findings may be generalisable across NICE. Naci et al. (2025) quantified the population‐health impact of 183 new drugs recommended by NICE during 2000–2020. They found that new drugs benefitted patients to the extent of generating 3·75 million additional years of full health but came at a considerable cost to other healthcare because reallocation of the extra expenditures on these new drugs to other NHS treatments/services could have generated 5·00 million additional years of full health (Naci et al. 2025, 56). Although Naci et al. (2025) do not explore underlying sociological aspects of NICE, their results could be consistent with a wider application by NICE of the sociological phenomenon of the permissive principle.

Insofar as our findings are generalisable, they suggest that the interests of the NHS and public health could be more adequately served by minimising the application of the permissive principle at NICE. Progress could be made by prohibiting manufacturer involvement in guideline development and severely restricting conflicts of interest among expert advisers in regulatory decision‐making about cost‐effectiveness assessment and guideline approval, given that manufacturers already have huge input from producing/providing the key clinical trials.

There is also a need to counteract neoliberal ideologies of patient choice and innovation—which we found to symbiotically reinforce the permissive principle in regulatory thinking through a tendency to give new drugs ‘the benefit of the doubt’ because they increased market ‘choice’—despite concerns that they were not good value for money (Charlton 2025). This could be advanced by a policy shift at the governmental level to assert the importance of robust cost‐effectiveness assessment in the interests of public health irrespective of ‘innovation’ and constructed ‘patient demand’ by rejuvenating the prominence of independent Expert Review Groups. That shift towards greater independence from commercial interests may also need to challenge the increasing neoliberal corporate bias of the regulatory state and address the independence of NICE more broadly as its funding from fees paid by pharmaceutical companies is rising sharply and now amounts to 21.3% of the Institute's income (NICE 2026). However, under the 2025 UK–US trade agreement, the UK government has committed NICE to raise its pharmaceutical cost‐effectiveness thresholds, which means that the NHS could pay up to 25% more per unit of health benefit from new drugs relative to other interventions elsewhere in the health system (Cross et al. 2026). Our study suggests that promoting pharmaceuticalisation of healthcare in this way is a misplaced priority. The UK government should resist such lobbying from industry and foreign governments by concentrating instead on encouraging NICE to develop cost‐effectiveness assessment processes robust enough to restrain further deployment of the permissive principle and optimise public health benefits.

## Author Contributions

**John Abraham:** conceptualization, investigation, writing – original draft, methodology, validation, visualization, writing – review and editing, formal analysis, project administration, data curation, supervision, resources. **Gowree Balendran:** conceptualization, investigation, writing – original draft, methodology, validation, visualization, writing – review and editing, software, formal analysis, project administration, data curation, resources.

## Ethics Statement

All interviews were completed with informed consent and approval from the Research Ethics Office, King's College London (Research Ethics Number MR/15/16‐14).

## Consent

The authors have nothing to report.

## Conflicts of Interest

The authors declare no conflicts of interest.

## Permission to Reproduce Materials From Other Sources

The authors have nothing to report.

## Data Availability

Data are available, some on publicly available websites.

## Endnotes

^1^
Hereafter, ‘stroke prevention’ refers to ‘prevention of stroke and systemic embolism in patients with NVAF’ and ‘patients’ refers to ‘NVAF/AF patients’.

^2^
Once approved as safe and efficacious by pharmaceutical product regulators, NICE assesses whether a drug is cost‐effective for use in England's NHS, making recommendations accordingly.

^3^
FDA (Food and Drug Administration) is the US drug‐product regulatory agency.

^4^
ICH is a pharmaceutical industry‐led international collaboration between government regulatory agencies and industry associations concerned with setting technoscientific standards for drug testing/evaluation (Abraham and Reed 2001).

^5^
Controversially, DOAC manufacturers used a composite‐*efficacy* endpoint counting *all* strokes (ischaemic and haemorrhagic), even though the purpose of anticoagulation therapy is to inhibit ischaemic strokes, whereas, strictly speaking, haemorrhagic strokes are potential *adverse effects* of anticoagulation, rather than indicators of efficacy (Connolly et al. 2010).

^6^
Dabigatran was approved at two doses in the UK/EU—110 mg for patients aged 80 years and over and 150 mg for under‐eighties.

^7^
In pharmaceutical evaluation, unblinded trials are generally regarded as less valid/reliable and more prone to bias than double‐blind trials.

^8^
Interview, former FDA reviewer of DOACs, 11 March 2019.

^9^
Interview, GDG member for CG180, London, 25 April 2019.

^10^
Interview, GDG member for CG180, London, 30 May 2019.
